# Tree species composition along environmental and disturbance gradients in tropical sub-montane forests, Tanzania

**DOI:** 10.1371/journal.pone.0282528

**Published:** 2023-03-08

**Authors:** Nandera Juma Lolila, Deo D. Shirima, Ernest William Mauya

**Affiliations:** 1 Department of Forest Engineering and Wood Sciences, College of Forestry, Wildlife and Tourism, Sokoine University of Agriculture, Morogoro, Tanzania; 2 Department of Ecosystems and Conservation, College of Forestry, Wildlife and Tourism, Sokoine University of Agriculture, Morogoro, Tanzania; 3 National Carbon Monitoring Centre, Sokoine University of Agriculture, Morogoro, Tanzania; University of Veterinary and Animal Sciences, PAKISTAN

## Abstract

Understanding the environmental and disturbance determinants of tree species dominance and community composition in an ecosystem, is important for informing management and conservation decisions, through maintaining or improving the existing forest composition and structure. This study was carried out to quantify the relationship between forest tree composition structure and environmental and disturbance gradients, in a tropical sub-montane forest of Eastern Usambara. Vegetation, environmental, and anthropogenic disturbance data for 58 plots across Amani and Nilo nature forest reserves were obtained. Agglomerative hierarchical cluster analysis and canonical correspondence analysis (CCA) were used to identify plant communities and analyze the influence of environmental variables and anthropogenic disturbances on tree species and community composition respectively. Four communities were identified and CCA results showed that the variation was significantly related to elevation, pH, Annual mean temperature, temperature seasonality, phosphorus nutrients and pressures from adjacent villages and roads. Likewise, environmental factors (climate, soil and topography) explained the most variation (14.5%) of tree and community composition in relation to disturbance pressure (2.5%). The large and significant variation in tree species and community patterns explained by environmental factors suggests a need for site-specific assessment of environmental properties for biodiversity conservation plans. Similarly, the intensification of human activities and associated impacts on natural environment should be minimized to maintain forest species composition patterns and communities. The findings are useful in guiding in policy interventions that focus on minimizing human disturbances in the forests and could aid in preserving and restoring the functional organization and tree species composition of the sub-tropical montane forests.

## Introduction

Tropical montane forests are characterized by complex ecological systems resulting from dramatic changes of physical and geographic properties [1] such as topography [2–5], including; slope [6], aspect [1,5], climate [7,8], soil [9] and their interactions [10–12] from the base to the summit of mountains [13] creating a multitude of microhabitats [3]. Tropical montane forests are the most unique, with high diversity of vegetation [14,15], but they are also among the most threatened ecosystems by anthropogenic activities [5,16,17]. Forest disturbances, particularly selective removal of trees [18], have impact on the species composition and dominance on mountainous landscape [11,19], particularly on the lower elevations [20]. The dynamics of interaction among individual species and their environmental and disturbances influence species composition through coexistence or exclusion [8]. Hence, plant species that respond equally to the same factors, will usually coexist giving rise to distinct patterns of plant species communities across the landscape [21,22] while plant species with restricted habitat requirements are most susceptible to extinction in the face of anthropogenic pressures [23,24].

Species composition and structure of the tropical montane forests vary across a wide range of ecological gradients [1,25]. African mountainous areas have large human population densities encircling and depending on forest resources for their livelihoods [26,27]. This results to high human impacts on plant species composition including resource utilization, road construction, and residential development [20], increasing the rates of deforestation and forest degradation due to over exploitation of resources [28]. The intensity of these disturbances is likely to decrease as the distance of the roads and villages from the boundary of the forest increases [29]. Tanzania government acknowledges deforestation as a major threat to biodiversity, and has committed to the establishment and management of protected areas (PAs), significantly reducing the rate of forest degradation and ecosystem fragmentation [30]. However, the effectiveness of PAs management and their ability to withstand anthropogenic pressures varies, depending on the nature of the anthropogenic activities that pose a threat to the Pas [31].

Several studies have demonstrated that disturbances, physical and geographic factors[25,32], shape the structure of the forest [20,33,34] reported that, the relationship between tree species determinants and community composition is complicated because it involves a wide range of factors and considerations that provide a variety of clues, which partially explain the relationship, and whose findings cannot be generalized to other ecosystems. Despite being classified as protected areas (PAs) [35], the scenic beauty and unique biodiversity of the Amani and Nilo nature forest reserves, along with the high degree of forest accessibility [36,37], makes them vulnerable to mass tourism pressure [38,39].On the other hand, there hasn’t been a thorough analysis of how the combined environment and anthropogenic pressures are affecting the tree species composition patterns in both nature reserves. Meanwhile, being able to predict how tropical forests will react to environmental change requires an understanding of how quickly the structure and composition of these forests recover and which factors govern these processes [1,40]. Hence, understanding the influence of environmental and disturbance factors on plant species composition [41] in an ecosystem, is a prerequisite in developing management plans [42], important for prioritizing conservation activities at local, regional and global scales [43]. Thus, this study analyzed forest tree species composition structure along varying environmental and disturbance gradients in Tropical Sub-montane forests, exploring (i) How does environmental (climate, topography and soil) and potential disturbance pressures explain the tree species and community composition in tropical sub-montane forests? (ii) How influential are environmental factors relative to disturbance pressures in explaining observed species composition?

## Materials and methods

### Study area

**Fig 1:**
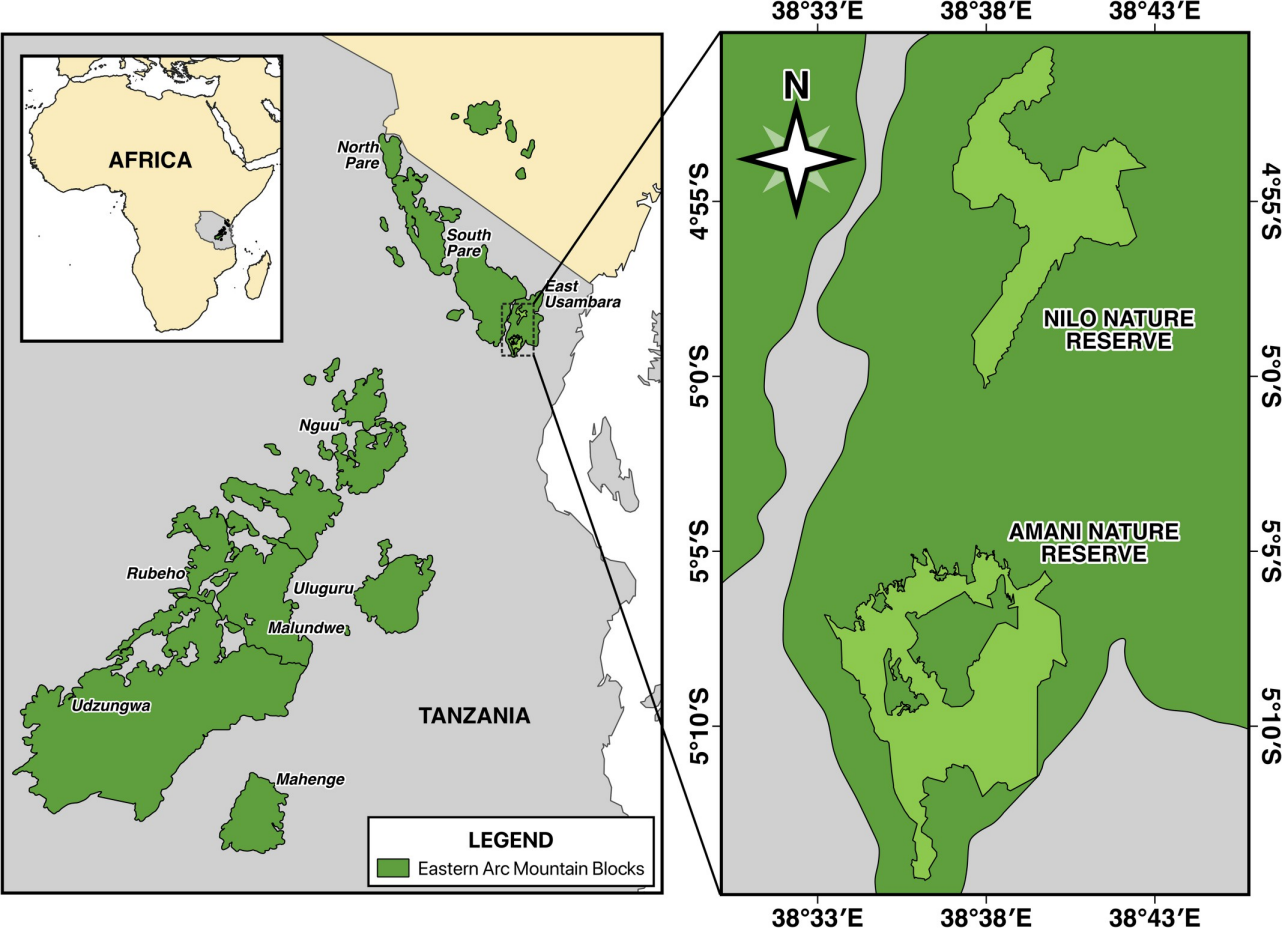
Map of the study area showing Eastern Arc mountains, Amani and Nilo nature forest reserve sites. The Eastern Arc layer source is from [35].

This study was conducted in Amani and Nilo nature forest reserve (NFR), located in Muheza, Mkinga and Korogwe Districts in Tanga Region of Tanzania. Amani nature Forest reserve (NFR) is located between 5°04’30” - 5°14’10” S and 38°30’34” - 38°40’06” E with an area of about 8360 ha. The elevation of ANFR is ranging from 190m-1130 m above the sea level [36,39]. NNFR is located between 4°50’ - 4°59’S and 38°37’ - 38°41’06” E, covering about 6025 ha (Fig 1). The location of NNFR is at an elevation range of 400 to 1506 m above the sea level. Both Amani NFR and Nilo NFR are confined to the East Usambara Mountains forests (EUMFs) located in eastern Tanzania and they are part of the Eastern Arc Mountains (EAM), which is a chain of mountains that stretches down the coast of East Africa from southern Kenya to southern Tanzania [36].

The rainfall distribution in EUMFs is bi-modal, peaking between March and May and between October and December [39]. Rainfall is greatest at higher altitudes and in the south-east of the mountains, increasing from 1,200 mm annually in the foothills to over 2,300 mm at higher altitudes [36]. The dry seasons are from June to August and January to March. The vegetation of these forests’ ranges includes lowland forest at 300 m on Eastern side, sub-montane forests and montane forests. Tree species composition varies considerably, but species such as *Khaya anthotheca*, *Milicia excelsa* are found in the lowlands and others such as *Myrianthus holstii*, *Albizia gummifera*, *Allanblackia stuhlmannii* and *Newtonia buchananii are* dominant at high altitudes [37,44].

### Sampling design

We utilized the existing systematic 450 by 900 m grids of east-west and north-south transect method in 1999–2000 by Frontier Tanzania in each of the two nature forest reserves [44]. The grids were intended for surveying the flora and fauna where 182 rectangular plots were established in Amani NFR and 122 rectangular plots of 20 x 50 were established in Nilo NFR.

In this study, we measured a sub -sample of 30 circular plots of 18 m radius within the Frontier grids in Amani NFR in 2017/18 as described in [45]. Sixteen (16) plots were on the exact position as in the frontier plots, with difference in shape and sizes only, 14 plots were established along the grid lines of Frontier plots and located exactly midway between two existing Frontier plots. Thus, the inter plot distance between our plots was 225 rather than 450m. The 30 plots covered an elevation range from 200 to 1000 m above sea level so that both the lowland forests (<800 m above sea level) and the sub-mountain forests (>800 m above sea level) were covered [37].

In Nilo NFR 28, circular plots of 18 m radius were established exactly on the same location with the existing Frontier plots. Same grids were used only with difference in plot size and shapes, thus the inter plot distance was 450m. We decided to use circular plots, because they are easier to establish in the field, and because they have one dimension (i.e., radius) that defines the plot boundary. Therefore, in total we had 58 field plots measured in the two nature forest reserves.

### Data collection

#### Field data

Existing plot coordinate’s location established by [45] were used to navigate to the center of field plots in Amani NFR using handheld GPS. Similarly, in Nilo NFR the plot coordinate’s locations as reported by [46] for the Frontier were used to locate the center position of the plots. Field data were collected in Dec 2017 and May 2018 in Amani NFR and Nilo NFR respectively. As stated above, circular plots with a radius of 18 m were established on each of the two-nature forest reserves. On each plot, all trees with diameter at breast height (*dbh*) greater or equal to 5cm were measured and recorded for *dbh*, scientific and local name. Plant materials were collected for further identification or confirmation at the Tanzania National Herbarium. A caliper was used to measure *dbh*, however for larger and trunked trees diameter tape was used.

#### Forest tree species composition and dominance

To determine the species composition of the forests, we summarized the species datasets into list of all species recorded and their abundance values.

The dominance of tree species was determined from the estimation of their species importance value index (SIVI).The Importance Values Index (IVI), indicates the ecological importance of a tree species and hence the dominance [20]. The Importance Value Index (IVI) for each species was computed as the sum of relative density, relative dominance and relative frequency of the species in each plot following [47].

#### Environmental data

To estimate the influence of environmental factors on tree species, we used information on topography, climate and soil factors.

Topographic factors including; elevation, slope and aspect were extracted from the raster layer derived from the SRTM 30 m-based DEM-USGS Earth Explorer (https://earthexplorer.usgs.gov/). A total of 58 points consisting of plot locations were imported into QGIS 3.22, where a spatial analyst tool of the QGIS was employed to obtain values of aspect, elevation and slope as well as the Topographic Position Index (TPI). Climatic data was downloaded from WorldClim site (https://www.worldclim.org) with a 30 arc seconds resolution and soil data was extracted from the Re-gridded Harmonized World Soil Database: ISRIC Data Hub (International Soil Reference and Information Centre) https://data.isric.org/geonetwork/srv/eng/catalog.search#/home. For each plot, values for all spatially interpolated climate and soil predictors were extracted using “raster package” in R software sampled using the coordinates of the plot center and averaged across plots per site (Table 1).

**Table 1:** The environmental and disturbance factors (Mean ± SD) within the tropical sub -montane forests of East Usambara, Tanzania. The bolded factors significantly explained species composition.

Factors	Variables	Codes	Mean	SD	Min	Max
Soil	**pH**	**pH**	**5.398**	**0.17**	**5.00**	**6.00**
	Organic carbon	OC	15.88	5.24	6.00	30.00
	Electrical conductivity	EC	0.67	0.30	0.13	1.49
	Cation exchange capacity	CEC	12.17	2.82	8.00	20.00
	Available water holding capacity	AWHC	26.83	1.39	24.00	30.00
	Bulk density	BD	1,364.48	54.55	1,260.00	1,490.00
	Potassium	K	**153.83**	**45.12**	**72.00**	**265.00**
	Total available Nitrogen	N	2,023.07	211.57	1,355.00	2,682.00
	Sodium	Na	217.71	42.79	179.00	381.00
	**Phosphorous**	**P**	**849.74**	**204.95**	**428.00**	**1,299.00**
Climatic	Mean Annual Temperature	MAT	21.82	1.42	20.14	24.48
	**Temperature seasonality**	**TS**	**164.31**	**12.07**	**140.53**	**186.86**
	**Mean Annual precipitation**	**MAP**	**1,337.71**	**147.32**	**1,073.00**	**1,589.00**
	Precipitation seasonality	PS	57.57	2.33	53.57	62.31
Topographic	Aspect	As	164.32	88.08	3.57	355.10
	**Elevation**	**El**	**704.36**	**301.46**	**222.65**	**1,120.00**
	Slope	**Sl**	**29.33**	**16.52**	**3.58**	**70.57**
	Topographical position Index	TPI	0.47	2.81	-4.74	9.44
Disturbance	**Distance to the road**	**Rd**	**6,378.83**	**4,355.10**	**19.08**	**12,385.68**
	**Distance to the villages**	Vl	1,561.55	634.05	461.22	3,384.29
	Distance to conservation offices	**HQDist**	**4,538.13**	**3,014.47**	**117.93**	**9,871.30**

#### Disturbance data

The degree of forest accessibility was included as proxy for anthropogenic disturbances [29,48]. Likewise, the presence of a conservation office building in the NFR was regarded as a factor for the decrease in intensity of human disturbances.

To quantify the influence of disturbance on tree species composition, we used distance from each field sampling plot to the nearest primary, secondary, or tertiary road, as well as the distance to the nearest city, village, or town and the distance from the plot to the conservators’ office. We obtained spatial data for roads and villages as shapefiles using road and settlement vector data from OpenStreetMap (http://download.geofabrik.de/africa/tanzania.html). The positions of the conservators’ offices were digitized from high resolution base-maps of the areas. The distances were obtained by calculating the Cartesian distance (in kilometers), using the gDistance function within the *rgeos* package of R statistical software. The function returns the minimum Cartesian distance between the two points (plots to the road or villages).

### Data analysis

#### Dominant tree species composition

We used detrended correspondence analysis (DCA) to detect the magnitude of the ecological gradient in our species composition matrix and hence the constrained ordination to be used [49,50]. The DCA results showed an axis length greater than 4.0 in both study sites, suggesting that the data is heterogeneous. We therefore used Canonical Correspondence analysis (CCA) [51] to determine the influence of environmental and disturbance on dominant tree species and community types.

Prior to CCA, we checked for collinearity among variables on the basis of Pearson correlation (r) and Variance Inflation Factor (VIF). Predictor variables with a VIF value greater than ten (10)) [52] and r > 0.7 [20] were considered to be high collinear [51] and were trimmed out from the list of predictors. Species abundance data were log-transformed to meet assumptions of multivariate normality [50]. Species that occurred in less than five plots were considered to have no major ecological significance (rare species) and were removed from the matrix [53]. Stepwise automatic forward selection was used to identify significant explanatory variables to be constrained in multivariate analysis [50]. The selected explanatory variables were then constrained against the tree species and community composition using CCA. The effect of the obtained explanatory variables on tree species composition in CCA model was determined using the Monte Carlo test with 999 unrestricted permutations [49] at 95% confidence interval.

#### Community composition

Similar communities were identified using cluster analysis. First, we computed the Bray–Curtis’s distance matrix using the abundance data and then, performed hierarchical Ward’s minimum variance clustering on the Bray–Curtis’s distance matrix. The decision on the number of clusters was based on Silhouette validation technique using *‘Nbclust package’* [54]. Indicator species analysis was performed to identify significant dominant species of the communities, using package *labdsv*. Then, community types were named after the two most dominant species [43] based on high synoptic cover abundance values (mean frequency multiplied by mean cover-abundance), following [49] (S1 Appendix).

Additionally, the community types obtained was subjected to ANOVA to describe environmental and disturbance differences among individual clusters. For the explanatory variables which were significant, pairwise differences tested among clusters; using Tukey HSD comparison was done.

#### Tree species composition variance partitioning

Explanatory variables that were significant in explaining variability in tree species composition were retained and considered for subsequent variance partitioning analysis. The significant explanatory variables were categorized into two main groups i.e., environmental and disturbance groups, then sub-grouped into four sets of climatic, topographic, edaphic and disturbance. We performed variance partitioning by partial CCA to determine independent and joint contributions of each group predictor variables to explain variation in species composition. The proportion of variation explained was given as the adjusted R^2^ of the explanatory variable in the CCA, as unbiased estimates of the variation [41]. DCA and CCA were performed by the ‘vegan’ package and variation partitioning with CCA by the ‘varpart’ package in the R software (v. 4.1.2) [21].

## Results

### Trees species and community structure

#### Tree species composition structure

A total of 2910 individuals were recorded belonging to 174 species, 128 genera, and 46 families. The most dominant families with a high number of species were: Leguminosae (28), Rubiaceae (19), Moraceaea (13), Malvaceae (11) and Sapotaceae (10). The most common genera with the highest number of trees were Albizia (5), Ficus (5) and Celtis (5). Also, the most dominant species, in terms of IVI were *Leptonychia usambarensis* K. Schum(151.8), *Cephalosphaera usambarensis* (Warb.) Warb. (114.6), *Antiaris toxicaria* Lesch (95.2) and *Maesopsis eminii Engl*. *(*85.7*)* (S1 Appendix).

**Fig 2:**
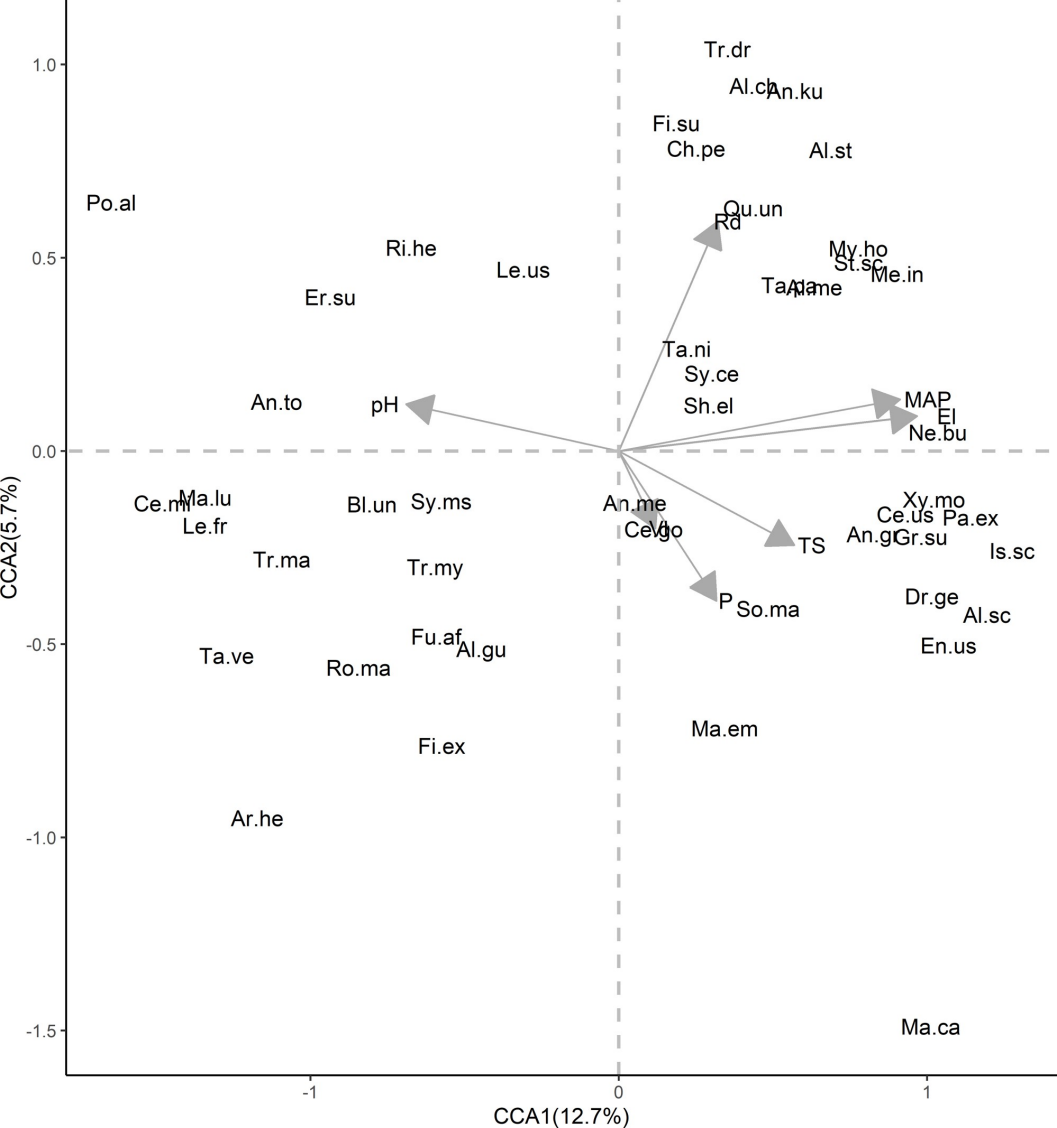
Canonical correspondence analysis diagram showing the ordination of dominant species represented and their correlation with environmental and disturbance variables in the sub-tropical montane forest plots. Canonical correspondence analysis biplot of Amani and Nilo FR species distribution (blue dots) based on tree species abundance. The gray axes vectors indicate the significant explanatory variables in their abbreviated form: El = Elevation, TS = Temperature seasonality, MAP = Mean Annual precipitation, P = Soil phosphorus, Rd = Distance to the road and Vl = Distance to the villages. We also used abbreviated species scientific names of each tree by using first two letters each from genus and species; *Al*.*ch = Alangium chinense (Lour*.*) Harms*, *Al*.*gu = Albizia gummifera (J*.*F*.*Gmel*.*) C*.*A*.*Sm*., *Al*.*me = Allophylus melliodorus Gilg ex Radlk*., *Al*.*st = Allanblackia stuhlmannii (Engl*.*) Engl*., *Al*.*sc = Alsodeiopsis schumannii (Engl*.*) Engl*., *An*.*ku = Annickia kummeriae (Engl*. *& Diels) Setten & Maas*, *An*.*gr = Anthocleista grandiflora Gilg*, *An*.*me = Antidesma membranaceum Müll*.*Arg*. *An*.*to = Antiaris toxicaria Lesch*., *Ar*.*he = Artocarpus heterophyllus Lam*, *Bl*.*un = Blighia unijugata Baker*, *Ce*.*go = Celtis gomphophylla Baker*, *Ce*.*mi = Celtis mildbraedii Engl*., *Ce*.*us = Cephalosphaera usambarensis (Warb*.*) Warb*., *Ch*.*pe = Chrysophyllum perpulchrum Mildbr*. *ex Hutch*. *& Dalziel*, *Dr*.*ge = Drypetes gerrardii Hutch*., *En*.*us = Englerodendron usambarense Harms*, *Er*.*su = Erythrophleum suaveolens (Guill*. *& Perr*.*) Brenan*, *Fi*.*ex = Ficus exasperata Vahl*, *Fi*.*su = Ficus sur Forssk*., *Fu*.*af = Funtumia africana (Benth*.*) Stapf*, *Gr*.*su = Greenwayodendron suaveolens (Engl*. *& Diels) Verdc*., *Is*.*sc = Isoberlinia scheffleri (Harms) Greenway*, *Le*.*fr = Lecaniodiscus fraxinifolia Baker*, *Le*.*us = Leptonychia usambarensis K*. *Schum*. *Ma*.*ca = Macaranga capensis (Baill*.*) Sim*, *Ma*.*em = Maesopsis eminii Engl*., *Ma*.*lu = Markhamia lutea (Benth*.*) K*.*Schum*., *Me*.*in = Mesogyne insignis Engl*., *My*.*ho = Myrianthus holstii Engl*., *Ne*.*bu = Newtonia buchananii (Baker) G*.*C*.*C*.*Gilbert & Boutiqu*, *Pa*.*ex = Parinari excelsa Sabine*, *Po*.*al = Pouteria alnifolia (Baker) Roberty*, *Qu*.*un = Quassia undulata (Guill*. *& Perr*.*) D*.*Dietr*. *Ri*.*he = Ricinodendron heudelotii (Baill*.*) Heckel*, *Ro*.*ma = Rothmannia manganjae (Hiern) Keay*, *Sh*.*el = Shirakiopsis elliptica (Hochst*.*) Esser*, *So*.*ma = Sorindeia madagascariensis Thouars ex DC*., *St*.*sc = Strombosia scheffleri Engl*., *Sy*.*ce = Synsepalum cerasiferum (Welw*.*) T*.*D*.*Penn*., *Sy*.*ms = Synsepalum msolo (Engl*.*) T*.*D*.*Penn*., *Ta*.*pa = Tabernaemontana pachysiphon Stapf*, *Ta*.*ve = Tabernaemontana ventricosa Hochst*. *ex A*.*DC*., *Ta*.*ni = Tarenna nigrescens (Hook*.*f*.*) Hiern*, *Tr*.*dr = Trichilia dregeana Sond*., *Tr*.*ma = Trilepisium madagascariense DC*., *Tr*.*my = Tricalysia myrtifolia S*.*Moore*, *Xy*.*mo = Xymalos monospora (Harv*.*) Baill*.

Our results show that, 28% of variation of dominant tree species composition was explained by the environmental and disturbance factors. The overall CCA model (χ^2^ = 0.58, P = 0.001), as well as the first two ordination axes were statistically significant (P = 0.001), showing that the observed patterns differed from a random relationship. The first two ordinations accounted for 15.9% of the total variation, having eigenvalues of 0.58 and 0.21 respectively. The first ordination axis was significantly correlated with elevation (El), mean Annual Precipitation (MAP) gradients, pH and Temperature seasonality (TS), while the second canonical axis showed strong significant correlations (P<0.05) with distance to the nearest road (Rd) and villages (Vl) (Fig 2).

The biplots indicated that, on axis 1, species like *Isoberlinia scheffleri* (Harms) Greenway, *Alsodeiopsis schumannii* (Engl.) Engl., *Parinari excelsa* Sabine have positive scores while species like *Pouteria alnifolia* (Baker) Roberty, *Celtis mildbraedii* Engl. and *Lecaniodiscus fraxinifolia* Baker have high negative scores and are highly significantly influenced by Elevation and Precipitation (MAP). On axis 2, species like; *Trichilia dregeana* Sond., *Alangium chinense* (Lour.) Harms and *Ficus sur* Forssk. had positive scores and are positively influenced by pressure of nearest roads (Fig 2).

#### Community composition

**Fig 3:**
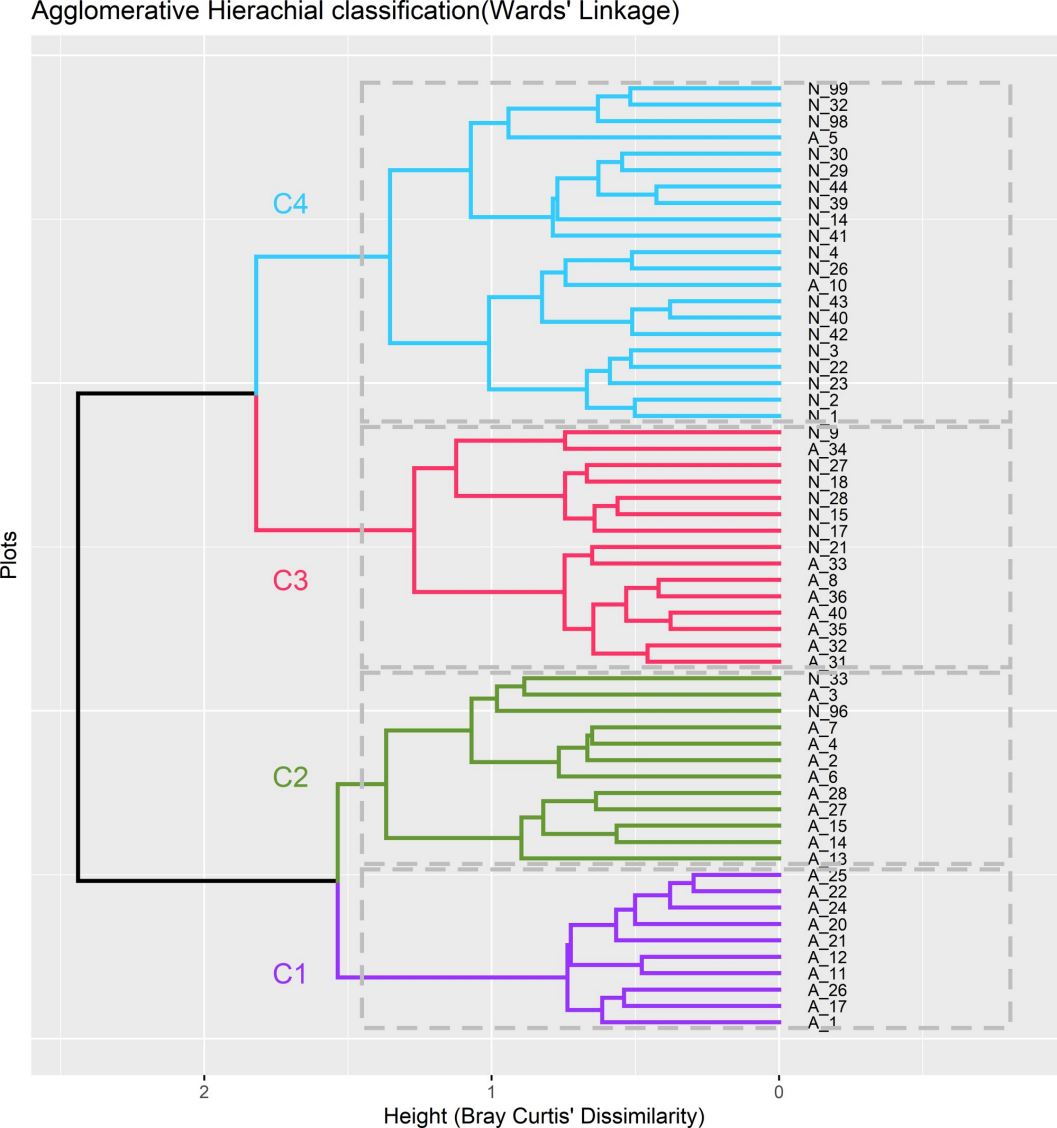
Dendrogram of hierarchical clustering using similarity ratio showing four community types in different sites (plot 1–59) of the study area (Ward’s method, Bray-Curtis distance) across Amani and Nilo nature forest reserve in sub-tropical East Usambara montane forest of Eastern Arc. The communities are named by using number of communities denoted as (Cn) and they are named after by two dominant species: C1 = *Tabernaemontana ventricosa-Leptonychia usambarensis*, C*2* = *Ficus sur- Myrianthus holstii*, C3 = *Artocarpus heterophyllus-Lecaniodiscus fraxinifolia* and C4 = *Isoberlinia schefflera- Sorindeia madagascariensis* communities (S1 Appendix).

We identified five distinct significant tree species communities (p < 0.001) in the study area (Fig 3). Most of the species were shared across communities; however each community is characterized by a distinct set of dominant species (S1 Appendix).

**Fig 4:**
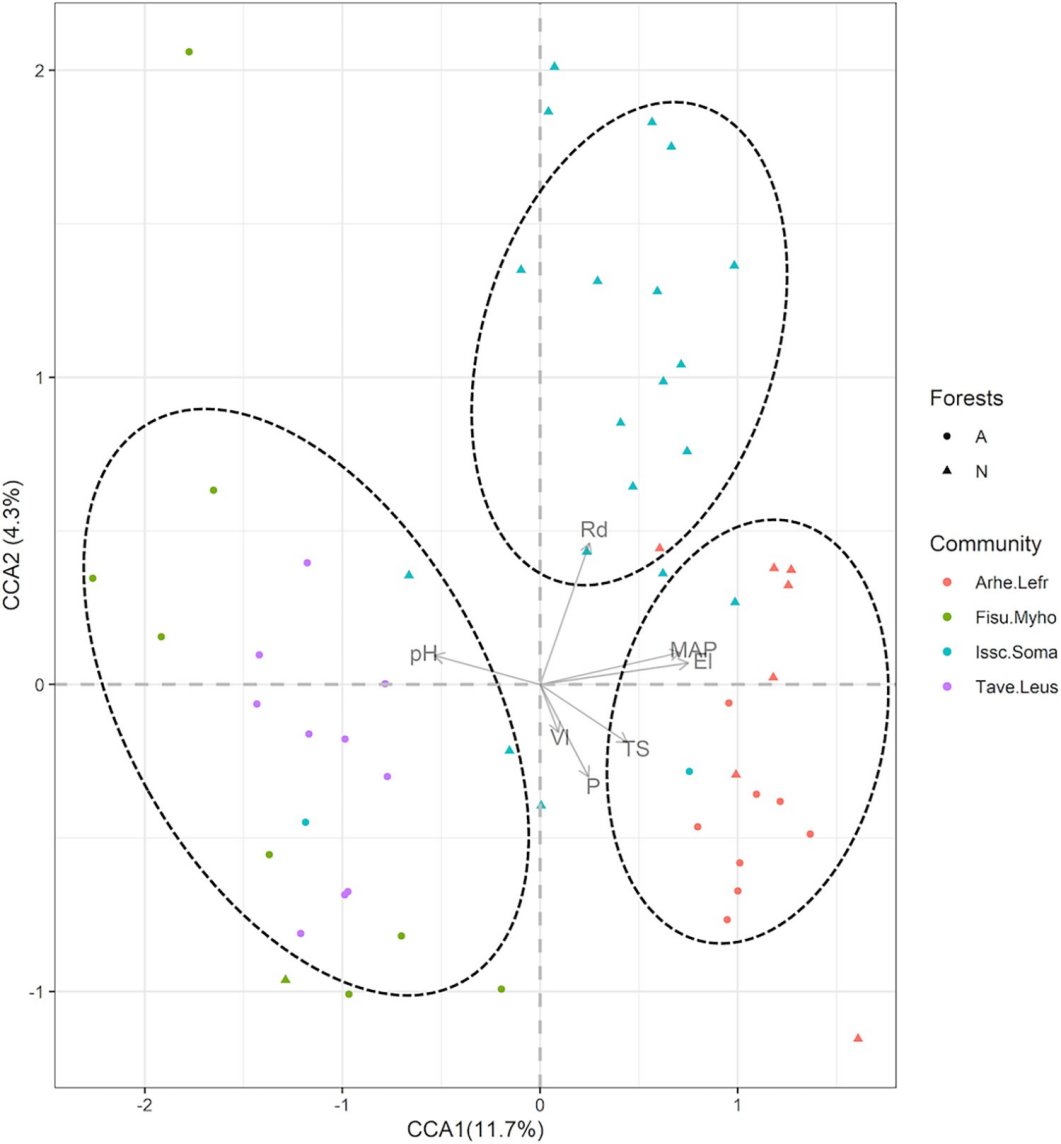
Canonical correspondence analysis diagram displaying the ordination of community types represented and their correlation with environmental and disturbance variables, indicating abundance relationships in sub-tropical East Usambara montane forest of Eastern Arc mountains. Communities: Tave.Leus = *Tabernaemontana ventricosa- Leptonychia usambarensis*, Arhe.Lefr = *Artocarpus heterophyllus-Lecaniodiscus fraxinifolia*, Fisu.Myho = *Ficus sur- Myrianthus holstii* and *Isoberlinia schefflera- Sorindeia madagascariensis* communities (S1 Appendix).

The community composition structure in the two forests were influenced by elevation (El), Phosphorous (P), pH, mean annual precipitation (MAP), temperature seasonality (TS), nearest distance to the road (Rd) and villages (Vl). The *Isoberlinia schefflera- Sorindeia madagascariensis* forest community is significantly influenced by pressures from nearest roads (P = 0.001), *Tabernaemontana ventricose-Leptonychia usambarensis and Ficus sur- Myrianthus holstii by* pH (P <0.05), while *Artocarpus heterophyllus—Lecaniodiscus fraxinifolia* forest community is influenced by mean annual precipitation, elevation and temperature seasonality (P <0.05) (Fig 4).

**Fig 5:**
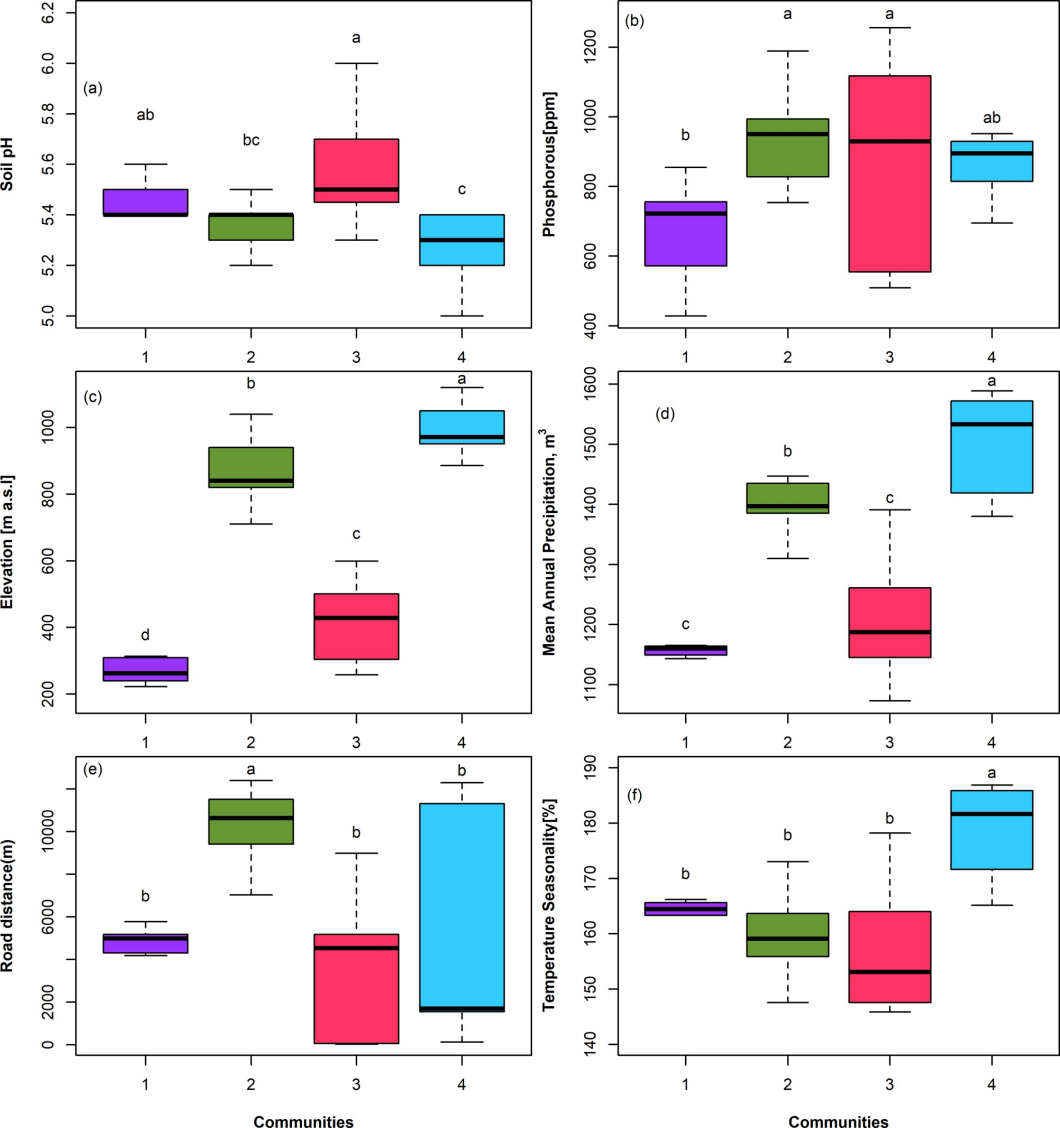
Boxplot showing difference among individual communities with respect to environmental and disturbance factors. The horizontal line crossing the box in the center represents the median; the box represents the 25^th^ and the 75^th^ percentiles. The vertical line outside the box represents the minimum and maximum values. Difference in letters (a, b, c, d) signifies that the communities are different in the specified factor. Communities: 1 = Tabernaemontana ventricosa-Leptonychia usambarensis, 2 = Ficus sur- Myrianthus holstii, 3 = Artocarpus heterophyllus-Lecaniodiscus fraxinifolia, 4 = Isoberlinia schefflera- Sorindeia madagascariensis.

Tree communities’ compositions are influenced by elevation (El), Phosphorous (P), pH, mean annual precipitation (MAP), temperature seasonality (TS), disturbance pressure from nearest road (Rd) and villages (Vl). The tree communities occupy significant different elevation gradients (P<0.05) (Fig 5A). Likewise, there is significance difference of the mean annual precipitation received by the communities with an exception of *Artocarpus heterophyllus—Lecaniodiscus fraxinifolia* (Arhe.Lefr) and *Tabernaemontana ventricosa-Leptonychia usambarensis* (Tave.Leus) communities (Fig 5B). There is no significant difference in the influence of disturbance pressures from the nearest villages (Vl) to tree communities (P > 0.05).

### Tree species composition variance partitioning

**Fig 6:**
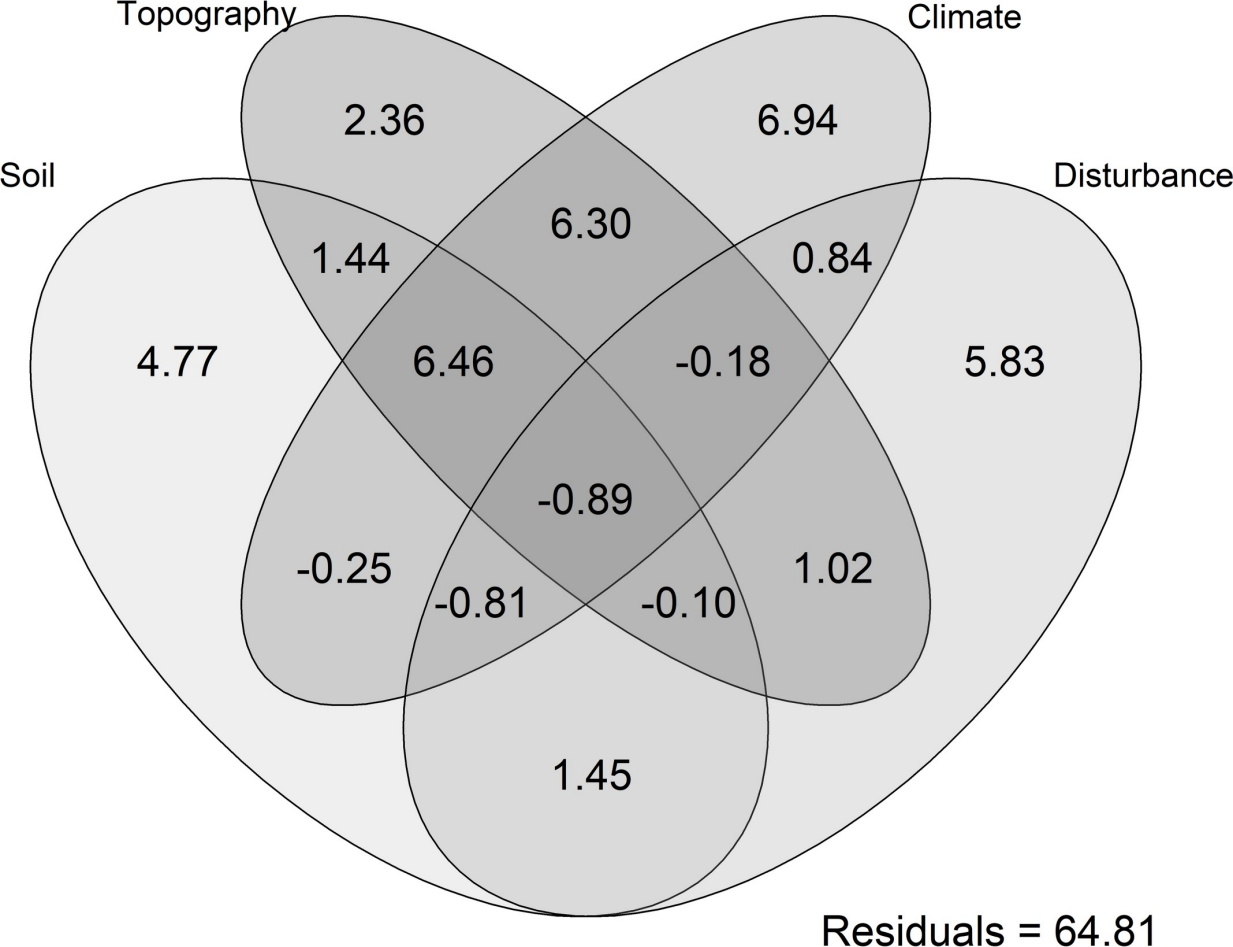
The Venn diagram showing variation partitioning results (partial CCA model) and the contribution of the four studied environmental and disturbance variable groups (i.e., climatic, soil, topography, and disturbance) that drive the plant species distribution. The proportion of variance in tree species composition explained by environmental and disturbance factors. The values represent adjusted R^2^ (%) of independent and shared effects by 4 the factors.

Results showed that environmental factors (14.5%) explained variability in tree species composition five times more than the potential disturbance factors (2.5%). Overall, the climate factors accounted for 6.95% of the variation in tree species composition (Table 2). The variation in species composition is also a result of interaction of multitude of these factors. Example; the interaction between climate and topography (6.3%) and climate, topography and soil (6.5%) explained high variation in species composition (Fig 6).

**Table 2:** CCA variation partitioning (%) on overall plant species composition of Tropical sub- montane forests of East Usambara, Northern Tanzania.

Effect and main variable	Covariables	Variation explained (%)	F-value	P-value
Total effect (Environmental and disturbance factors)		28.90	8.18	0.001
Partial effect				
Environmental	Anthropogenic pressure	14.50	1.93	0.001
Disturbance	Environmental	2.50	2.75	0.001
Soil	Climate, Topography and Anthropogenic pressure	4.80	1.63	0.009
Topography	Soil, Climate and Anthropogenic pressures	2.30	1.59	0.030
Climate	Soil, Topography and Anthropogenic pressure	6.95	2.01	0.001
Anthropogenic pressure	Soil, Topography and Climate	5.80	1.80	0.001

## Discussion

### Tree species community structure

Divergent distribution patterns of species and community composition of mountain ecosystems are influenced by dramatic changes in environmental conditions over short altitudinal distance [33,49,55,56]. Nevertheless, human exploitation may be imposing constraints that result in environmental modification [57] causing a shift in species range [58] and thus influencing species composition [59]. Our study found that tree species and community patterns are determined by climate, topography, soil and disturbance pressures that exhibit heterogeneity over the sub-montane forest of East Usambara.

Our results showed that elevation significantly influences differences in species communities, similar to mean annual precipitation and temperature seasonality as shown in the CCA (Fig 5), suggesting correlations among topographic, elevation and precipitation [60] perhaps due to adiabatic decrease of temperature with elevation [61] and elevation-dependent wetting (EDWE) [62] phenomenon. Nevertheless, elevation is linked to variation in humidity [59] and soil moisture [9], that may indirectly affect tree nutrient availability [43] and other factors, which affecting tree species establishment, and their community composition [6,63]. Several studies have shown that elevation-temperature relationship significantly influenced the physiological attributes of plant species [59].

Our study shows similar biogeographical patterns of communities where, *Artorcarpus heterophyllus-Lecaniodiscus franxinifolia* and *Isoberlinia schefflera-Sorindeia madagascariensis* communities, occupying lower to mid- elevation have higher species richness in comparison to other communities occupying high elevations. Studies suggest that, middle to low elevation zones have relatively mild climate and favorable nutritional conditions [64], meaning which means high chance for any species’ growth and survival. Many of the species in the forests are limited by extremities in temperatures [24,59] as well as precipitation [65]. Seasonal variation in temperature across most tropical forests, maybe minor [66]⁠, but recent studies suggest that small changes in temperature are likely to affect species distribution patterns according to their species physiological drought tolerance [65,67,68]. Climatic conditions can thus exert selective pressure on composition species [8], given that some tree species may not prefer areas of high elevation because of low temperatures [55,69]. Thus, elevation can be used as a proxy for understanding tree species adaptation to climate change [56].

Furthermore, we found that species and community composition are significantly explained by the variation of pH and phosphorous (P) nutrients (Figs 2 and 4). Our study sites occupy areas of acidic to slightly acidic (i.e. pH between 5 and 6). Similar to [70], our results suggests that soil pH is a substantial, driver of species composition and distribution in East Usambara. Others studies, also show that soil pH has the ability to influence tree species nutrient uptake and their ecological behavior at different localities. [70–72]. Moreover, we found that soil phosphorus significantly (P < 0.05) influences the variability of the tree species and communities. Regardless of phosphorus being important for regulating plant primary productivity, and other biological processes such root allocation and growth [73–75], it is limited to plants due to its low solubility and the sorption processes in soils [72]. Nevertheless, soil P availability can be affected by direct and indirect climatic processes through sorption and desortion [76]. The relationship between soil properties and climate can thus never be inseparable [9,22] as well as topography, as there’s evidently reports of elevation-climate dependency [43,59]. This suggests that any huge changes to the ecosystem may have knock- on effects on the whole forest system.

We found that disturbance had a significant influence on species composition in sub-tropical forest of East Usambara. *Isoberlinia schefflera-Sorindeia madagascariensis* communities forest community was influenced by disturbance factors and is the richest (59 species), while *Tabernaemontana ventricose-Leptonychia usambarensis* forest community is less affected by disturbances and had the least tree species richness (24 species). The patterns of species richness, corresponds to the intermediate disturbance hypothesis, which suggests that local species richness is maximized when ecological disturbance is neither too rare nor too frequent and not too intense [77]. We also found that a dominant species like *Maesopsis eminii* Engl, a light -demanding pioneer tree species, was influenced by disturbance i.e., adjacent to villages (Fig 2). It is evidently supported that disturbance pressures to the forests may exert effects on the natural environment, thus changing the site condition and species habitat, enabling conditions for new species to emerge and/or diminish the existing species [33]. Light demanding species like *M*. *eminii* Engl have mostly been in high proportion in disturbances gaps, due to available maximum light necessary for triggering their establishment and growth [78], but responses to disturbance pressures vary among tree species, depending on their ability to colonize and to compete [79].

### Tree species composition variance partitioning

The results of partial CCA (pCCA) revealed that species composition was defined best by climate and disturbance factors, with most of the variation being explained by the climate variable similar to [79]. Our results support the argument that mountain tree communities are sensitive to climate and, thus, able to reveal its effects sooner than others [56]. Variation of species is also explained by interaction of factors, including: Climate, topography and soil (6.5%), as well as climate and topography (6.3%) (Fig 6). This confirms that species composition is a result of combination of multiple factors [20]. Although the influences among the environmental and disturbance variables covaried, neither the effect of one variable was entirely nested within the other (% variation of each individual variable >1). This indicates that the individual contribution of each factor has important and independent effects [21] on community structure in a wide variety of tropical forest communities [74].

Next to climate (mean annual precipitation and temperature seasonality), disturbance explained the most variability of species composition. We expect that the protected reserves are supposed to be free from outside human intrusions, but the protective efficiency of the protected forest is still in question, as anthropogenic activities continue to pose threats to the forest reserves [30,31]. Several studies [19,33,79] have recognized the importance of assessing human activities in shaping the floristic composition of the forests. Our study used proxy values of nearest distance to the roads and villages, which indicated that human-induced threats such as. selective logging, [26,38], had significant influence on the variability in species and community composition. Chronic disturbance generated by anthropogenic activities normally gives rise to alterations in habitat conditions through the modification of physical structure of the forest [18].

## Conclusion

Understanding the influence of environmental and disturbance factors on tree species dominance and community composition in an ecosystem, is important to inform conservation of existing forest structures. Our findings suggest that there is a strong relationship among forest structure, environmental and disturbances factors. The large and significant variation in tree species and community patterns explained by environmental factors suggests a need for site-specific assessment of environmental properties for biodiversity conservation plans. We also showed that, human induced disturbances from easy road access and villages near to forests may influence tree species composition. The effects of site condition modification as a result of human induced disturbance vary with location and existence of flora composition depending on the ability of tree species to adapt either at small scale or massive changes. Human induced disturbances should be discouraged in order to maintain species composition and encourage continuous monitoring and sustainable use of forest community structure. Likewise, understanding tree species composition and community structure patterns and their underlying environmental factors is worthwhile when considering conservation of montane sub-tropical forests. Thus, our findings may be useful for formulation of forest restorationpolicy interventions to sustain the tree species composition and the functioning in the subtropical montane forests of Eastern Usambara Mountain and similar ecosystems elsewhere.

## Supporting information

S1 Appendix List of dominant species in Sub-tropical forest of Eastern Arc montane forests, Tanzania. The bolded species are indicator species with highest indicator values which were used for naming the communities in Sub-tropical East Usambara montane forest of Eastern Arc mountains, Tanzania. IVI = Importance value index and CCA1 and CCA2 = Canonical correspondence analysis showing values for species along axis 1 and 2.
(PDF)
