# Supplementary material for: Tree species composition along environmental and disturbance gradients in tropical sub-montane forests, Tanzania
Source: PLoS One. 2023 Mar 8;18(3):e0282528. doi: 10.1371/journal.pone.0282528 (PMC9994703; doi:10.1371/journal.pone.0282528)
Supplement: S1 Appendix — The bolded species are indicator species with highest indicator values which were used for naming the communities in Sub-tropical East Usambara montane forest of Eastern Arc mountains, Tanzania. IVI = Importance value index and CCA1 and CCA2 = Canonical correspondence analysis showing values for species along axis 1 and 2. (PDF) [file pone.0282528.s001.pdf]

***S1 Appendix*: List of dominant species in Sub-tropical forest of Eastern Arc montane forests.** The bolded species are indicator species with highest indicator values which were used for naming the communities in Sub-tropical East Usambara montane forest of Eastern Arc mountains, Tanzania. IVI = Importance value index and CCA1 and CCA2 = Canonical correspondence analysis showing values for species along axis 1 and 2.

Scientific names	*Species code*	*IVI*	*CCA1*	*CCA2*	*Community type*	*Indicator value*
Alangium chinense (Lour.) Harms	*Al.ch*	*9.50*	*0.40*	*0.79*	*2*	*0.53*
Albizia gummifera (J.F.Gmel.) C.A.Sm.	*Al.gu*	*5.87*	*-0.40*	*-0.43*	*1*	*0.43*
Allophylus melliodorus Gilg ex Radlk.	*Al.me*	*10.91*	*0.58*	*0.35*	*2*	*0.48*
Allanblackia stuhlmannii (Engl.) Engl.	*Al.st*	*6.27*	*0.63*	*0.65*	*4*	*0.58*
Alsodeiopsis schumannii (Engl.) Engl.	*Al.sc*	*64.46*	*1.09*	*-0.35*	*4*	*0.61*
Annickia kummeriae (Engl. & Diels) Setten & Maas	*An.ku*	*7.27*	*0.52*	*0.78*	*2*	*0.45*
Anthocleista grandiflora Gilg	*An.gr*	*8.61*	*0.75*	*-0.18*	*4*	*0.53*
Antidesma membranaceum Müll.Arg.	*An.me*	*5.91*	*0.05*	*-0.11*	*4*	*0.27*
Antiaris toxicaria Lesch.	*An.to*	*10.66*	*-1.01*	*0.11*	*1*	*0.75*
Artocarpus heterophyllus Lam	***Ar.he***	***18.54***	***-1.07***	***-0.79***	***3***	***0.67***
Blighia unijugata Baker	*Bl.un*	*95.24*	*-0.73*	*-0.11*	*1*	*0.64*
Celtis gomphophylla Baker	*Ce.go*	*7.79*	*0.10*	*-0.17*	*2*	*0.38*
Celtis mildbraedii Engl.	*Ce.mi*	*9.77*	*-1.35*	*-0.11*	*3*	*0.43*
Cephalosphaera usambarensis (Warb.) Warb.	*Ce.us*	*7.35*	*0.85*	*-0.14*	*4*	*0.76*
Chrysophyllum perpulchrum Mildbr. ex Hutch. & Dalziel	*Ch.pe*	*8.13*	*0.23*	*0.65*	*2*	*0.40*
Drypetes gerrardii Hutch.	*Dr.ge*	*17.46*	*0.92*	*-0.31*	*4*	*0.57*

Englerodendron usambarense Harms	*En.us*	*47.28*	*0.97*	*-0.42*	*4*	*0.47*
Erythrophleum suaveolens (Guill. & Perr.) Brenan	*Er.su*	*15.01*	*-0.85*	*0.33*	*3*	*0.37*
Ficus exasperata Vahl	*Fi.ex*	*11.30*	*-0.52*	*-0.64*	*2*	*0.33*
Ficus sur Forssk.	***Fi.su***	***114.63***	***0.17***	***0.71***	***2***	***0.70***
Funtumia africana (Benth.) Stapf	*Fu.af*	*11.10*	*-0.54*	*-0.40*	*1*	*0.70*
Greenwayodendron suaveolens (Engl. & Diels) Verdc.	*Gr.su*	*10.79*	*0.89*	*-0.18*	*4*	*0.59*
Isoberlinia scheffleri (Harms) Greenway	***Is.sc***	***8.85***	***1.16***	***-0.21***	***4***	***0.84***
Lecaniodiscus fraxinifolia Baker	***Le.fr***	***6.53***	***-1.22***	***-0.16***	***3***	***0.64***
Leptonychia usambarensis K. Schum.	***Le.us***	***10.13***	***-0.28***	***0.39***	***1***	***0.92***
Macaranga capensis (Baill.) Sim	*Ma.ca*	*9.95*	*0.92*	*-1.24*	*2*	*0.54*
Maesopsis eminii Engl.	*Ma.em*	*7.69*	*0.31*	*-0.60*	*4*	*0.63*
Markhamia lutea (Benth.) K.Schum.	*Ma.lu*	*7.16*	*-1.22*	*-0.10*	*3*	*0.38*
Mesogyne insignis Engl.	*Me.in*	*21.74*	*0.82*	*0.38*	*4*	*0.58*
Myrianthus holstii Engl.	***My.ho***	***79.49***	***0.71***	***0.44***	***2***	***0.62***
Newtonia buchananii (Baker) G.C.C.Gilbert & Boutiqu	*Ne.bu*	*12.40*	*0.94*	*0.04*	*4*	*0.66*
Parinari excelsa Sabine	*Pa.ex*	*34.04*	*1.04*	*-0.14*	*4*	*0.43*
Pouteria alnifolia (Baker) Roberty	*Po.al*	*20.21*	*-1.50*	*0.54*	*3*	*0.57*
Quassia undulata (Guill. & Perr.) D.Dietr.	*Qu.un*	*151.76*	*0.40*	*0.52*	*2*	*0.36*
Ricinodendron heudelotii (Baill.) Heckel	*Ri.he*	*23.43*	*-0.61*	*0.44*	*1*	*0.42*
Rothmannia manganjae (Hiern) Keay	*Ro.ma*	*85.65*	*-0.77*	*-0.47*	*1*	*0.64*

Shirakiopsis elliptica (Hochst.) Esser	*Sh.el*	*6.05*	*0.27*	*0.10*	*4*	*0.36*
Sorindeia madagascariensis Thouars ex DC.	***So.ma***	***6.94***	***0.44***	***-0.34***	***4***	***0.77***
Strombosia scheffleri Engl.	*St.sc*	*16.38*	*0.71*	*0.41*	*4*	*0.52*
Synsepalum cerasiferum (Welw.) T.D.Penn.	*Sy.ce*	*10.67*	*0.27*	*0.17*	*2*	*0.27*
Synsepalum msolo (Engl.) T.D.Penn.	*Sy.ms*	*11.38*	*-0.52*	*-0.11*	*1*	*0.76*
Tabernaemontana pachysiphon Stapf	*Ta.pa*	*52.80*	*0.50*	*0.36*	*2*	*0.47*
Tabernaemontana ventricosa Hochst. ex A.DC.	***Ta.ve***	***29.60***	***-1.16***	***-0.44***	***1***	***0.94***
Tarenna nigrescens (Hook.f.) Hiern	*Ta.ni*	*21.56*	*0.20*	*0.22*	*2*	*0.38*
Trichilia dregeana Sond.	*Tr.dr*	*30.30*	*0.32*	*0.87*	*2*	*0.45*
Trilepisium madagascariense DC.	*Tr.ma*	*7.16*	*-0.99*	*-0.23*	*1*	*0.57*
Tricalysia myrtifolia S.Moore	*Tr.my*	*9.88*	*-0.54*	*-0.25*	*1*	*0.41*
Xymalos monospora (Harv.) Baill.	*Xy.mo*	*20.90*	*0.93*	*-0.10*	*4*	*0.48*
